# Impaired Baroreflex Sensitivity after Bilateral Convulsive Seizures in Patients with Focal Epilepsy

**DOI:** 10.3389/fneur.2017.00210

**Published:** 2017-05-18

**Authors:** Kevin G. Hampel, Christian E. Elger, Rainer Surges

**Affiliations:** ^1^Department of Epileptology, University Hospital Bonn, Bonn, Germany; ^2^Multidisciplinary Epilepsy Unit, Neurology Service, University Hospital La Fe, Valencia, Spain

**Keywords:** epileptic seizures, autonomic nervous system, systemic blood pressure, heart rate, mortality, sudden unexpected death in epilepsy

## Abstract

**Background:**

Sudden unexpected death in epilepsy (SUDEP) is probably due to an autonomic failure in the early postictal phase after bilateral convulsive seizures (BCS) in the majority of cases. The baroreflex sensitivity (BRS) is an established and reliable biomarker of autonomic function and sudden cardiac death.

**Objective:**

To investigate whether postictal BRS depends on seizure type.

**Methods:**

Beat-to-beat systemic blood pressure and heart rate were continuously and non-invasively recorded with the ccNexfin^®^ device in patients with focal epilepsy undergoing video-EEG monitoring. BRS was calculated using the sequence as well as the spectral method. A random mixed linear model was applied to analyze the influence of seizure type on BRS during three different time periods of 15-min length each (interictal, preictal, and postictal). In addition, the possible effects of other factors (hypertension, hemispheric lateralization of ictal activity, epilepsy type, body position, vigilance state) were explored. Data are given as median with interquartile range.

**Results:**

A total of 26 seizures of 26 patients were analyzed. In BCS (*n* = 7), BRS significantly dropped from a preictal value of 15.0 ms/mm Hg (13.0–19.4) and an interictal value of 15.6 ms/mm Hg (12.0–20.4) to 3.1 ms/mm Hg (2.7–10.5) during the postictal period (*p* < 0.0001) according to the sequence method. This finding was replicated with the spectral method. In contrast, focal seizures (*n* = 19) did not lead to significant alterations of BRS in the postictal phase.

**Conclusion:**

Postictal BRS depends on the seizure type and is markedly impaired after BCS. The present study provides further evidence for a disturbed autonomic function following BCS. These findings might be related to cardiovascular failure in the context of SUDEP.

## Introduction

Sudden unexpected death in epilepsy (SUDEP) is the most frequent directly epilepsy-related cause of premature death in people with epilepsy (1). Except for stroke, no other neurologic disorder leads to more loss of potential patient years than SUDEP (2). The current knowledge points toward an early postictal autonomic failure after bilateral convulsive seizures (BCS) as the cause of death in most SUDEP cases (3). Recently, we found that heart rate (HR) and systemic blood pressure (BP) are differentially modulated in the early postictal phase after BCS, suggesting possible autonomic imbalance (4). While HR was elevated by 75% 2 min after BCS-cessation, the mean arterial BP was only increased by 15% and even dropped slightly below the preictal values 5 min after seizure termination. Because systemic BP is a critical hemodynamic factor, it is tightly regulated to maintain adequate perfusion of vital organs like the heart and brain (5). The baroreflex regulates short- and long-term BP modulation and represents one of the most reliable indicators for autonomic function (6). Baroreflex dysfunction was previously found in patients with temporal lobe epilepsy in the interictal period as compared to healthy controls (7). However, seizure-related changes of the baroreflex function have not been reported yet. Here, we investigated whether postictal baroreflex sensitivity (BRS) depends on the seizure type.

## Patients and Methods

### Patients

Adult patients aged 18 years or older who were evaluated by video-EEG-monitoring for epilepsy surgery or differential diagnosis were prospectively enrolled from September 2013 to December 2015 in the Department of Epileptology, University Hospital Bonn (Germany). Data on seizure-related HR and systemic BP of included patients were previously published (4). Epilepsies and seizures were classified according to the revised International League Against Epilepsy terminology (8).

### Data Recording and Processing

Patients underwent conventional scalp EEG recordings according to the 10–20 system or invasive presurgical monitoring with intracranial electrodes according to the results of prior non-invasive video-EEG telemetry (Micromed S.p.A., Mogliano Veneto, Italy). In addition, pulse rate, oxygen saturation (LNCS DC-I^®^ reusable sensor, Masimo, Irvine, CA, USA), and arterial BP were continuously and non-invasively recorded from beat-to-beat using the ccNexfin^®^ device (BMEYE, Amsterdam, the Netherlands) for up to 8 h a day (maximum approved time span for the device applied at a single finger). The exact methodology for the data recording was described previously (4). For each seizure, consecutive values of systolic BP and HR obtained by the ccNexfin^®^ device at three different time periods (interictal, i.e., starting 5 min after the beginning of the recordings or starting 20 min before the end of the recording; preictal, i.e., up to the last 2 min before the seizure start; postictal, i.e., starting 2 min after seizure end) of 15 min duration each were considered. The period length of 15 min was chosen, because the sequence method for BRS requires a time interval of at least 15 min to obtain reliable results (9). Seizures with (i) overlapping time intervals of interictal and preictal or interictal and postictal intervals or (ii) seizures with periictal time intervals below 15 min were excluded from the analysis. We assessed BRS with two different methods, the sequence and the spectral method. The modified sequence method was applied with the following settings: the threshold for change in R wave-to-R wave interval (R-R interval) 4 ms; a zero time shift between the systolic PB pulse and R-R interval; and a correlation coefficient threshold of 0.8 between systolic PB and R-R interval sequences and the whole average of negative and positive slopes (10, 11). The modified spectral method was applied using the average of the whole low frequency band (12–14).

### Statistical Analysis

A linear random mixed effect model was applied with restricted maximum likelihood to estimate the effect of time interval (interictal, preictal, and postictal) and seizure type (BCS, FS) on BRS (15). Since the BRS calculated with the sequence and the spectral method was right skewed, we used the log-transformed BRS as the outcome variable for the analysis. As a fixed effect we entered time interval and seizure type, as well as possible influencing factors including hemispheric lateralization of ictal activity (left, right, or bilateral), epilepsy type (temporal lobe epilepsy or extra-temporal lobe epilepsy), preictal vigilance state (awake or asleep), preictal body position (laying or sitting), circumstances (spontaneous or triggered), hypertension (taking antihypertensive drugs or not), and postictal generalized EEG suppression (PGES present or not). As random effects, we included random intercepts for patients to account for non-independence in the data. We used backward selection to find the best model (16). Normality of residuals and random effects of the final model were validated by visual inspection and the Shapiro–Wilk test. Data are given as median with the interquartile range (IQR) or as geometric mean with corresponding 95% confidence interval. *p*-values ≤0.05 were considered significant. We used simultaneous inference procedures to adjust the *p*-values with the Holm–Bonferroni method to further test the comparison between the different time intervals of FS and BCS (17). The statistical analysis and the graphs were performed using R version 3.3.0 with the packages dplyr version 0.4.3, multcomp version 1.4.5, and ggplot2 version 2.1.0 (R Foundation for Statistical Computing, Vienna, Austria).

## Results

Forty-five seizures were considered for the analysis, but 19 seizures had to be excluded because time intervals were shorter than the required time length of 15 min. Finally, a total of 26 seizures of 26 patients with drug-resistant focal epilepsy met the inclusion criteria and were analyzed. Table 1 displays the clinical summary data of these patients (further details on patient and seizure characteristics are provided in Tables 2 and 3). Several factors and potential confounders were tested for possible associations and interactions with BSR. Since preictal vigilance state, preictal body position, circumstances, hypertension, epilepsy type, and hemispheric lateralization appeared to have no significant effect on BSR, the final model for both methods (sequence and spectral method) included time interval, seizure type, and the interaction between time interval and seizure type. Tables 4 and 5 display the summary statistics of the two models.

**Table 1:** **Summary of clinical characteristics**.

Characteristics	BCS (*n* = 7)	FS (*n* = 19)
Age (median, IQR)	33.4 (27.7−44.6)	39.5 (32−43.5)
Female (no.)	5	7
Age at disease onset (median, IQR)	20 (10−23)	19 (9.5−29)
Localization of seizure onset (no.)		
Temporal	5	13
Extra-temporal	2	6

*IQR, interquartile range; BCS, focal seizure evolving to bilateral convulsive seizure; FS, focal seizure*.

**Table 2:** **Clinical characteristics of patients with BCS and FS**.

Patient no.	Sex	Age (years)	Age at onset (years)	Type of recorded seizure	Epilepsy type (hemispheric lateralization)	EEG (interictal)	Cerebral MRI
1	F	31.9	13	FS	FLE (U)	No epileptic activity	Normal
2	F	42.8	37	FS	TLE (L)	Normal	Gliosis temporal (L)
3	F	51.6	6	BCS	TLE (R)	Temporal-occipital (R)	Hippocampal sclerosis (B)
4	F	44.2	34	FS	FLE (R)	Frontal (B)	No epileptic lesion
5	F	42.1	20	BCS	TLE (R)	Temporal (B)	Hippocampal sclerosis (R)
6	M	23.7	19	FS	TLE (R)	Temporal (L)	Gliosis occipital (R)
7	M	32.1	11	FS	TLE (L)	No epileptic activity	Normal
8	M	47.0	31	BCS	TLE (R)	Temporal-occipital (R)	Hippocampal sclerosis (R)
9	M	32.7	22	FS	TLE (R)	Generalized	Tumor temporal (R)
10	F	29.1	25	BCS	TLE (R)	Temporal (B)	No epileptic lesion
11	M	48.2	17	FS	TLE (L)	Temporal (L)	Tumor occipital (R)
12	F	33.4	21	BCS	Temporal-parietal (L)^a^	Frontal-temporal-central (L)	No epileptic lesion
13	F	26.2	14	BCS	TLE (R)	Temporal (L)	Heterotopia lateral ventricle (R)
14	M	25.8	1	FS	TLE (R)	Temporal (R)	Hippocampal sclerosis (R)
15	M	38.8	31	FS	FLE (L)	Normal	Normal
16	F	39.5	16	FS	TLE (L)	Temporal (B)	No epileptic lesion
17	F	19.3	7	FS	FLE (L)	Normal	Focal cortical dysplasia cingulate cortex (L)
18	M	40.4	27	FS	TLE (L)	Temporal (L)	Normal
19	M	39.6	30	FS	TLE (L)	Normal	Multiple lesions
20	M	18.0	4	BCS	FLE (L)	Frontal (R)	Focal cortical dysplasia frontal (R)
21	M	61.9	22	FS	TLE (R)	Normal	No epileptic lesion
22	M	20.9	7	FS	FLE (L)	Frontal (L)	Normal
23	F	55.3	2	FS	TLE (R)	Temporal (R)	Hippocampal sclerosis (R)
24	M	40.3	31	FS	TLE (R)	Temporal (R)	Hippocampal sclerosis (R)
25	F	46.9	8	FS	FLE (L)	Generalized	Extensive focal cortical dysplasia (L)
26	M	38.5	28	FS	TLE (L)	Temporal (L)	Hippocampal sclerosis (L)

*B, bilateral; BCS, bilateral convulsive seizures; FLE, frontal lobe epilepsy; F, female; FSs, focal seizures; L, left; M, male; R, right; TLE, temporal lobe epilepsy; U, undefined*.

*^a^According to intracranial EEG monitoring extended seizure onset zone left temporal-parietal*.

**Table 3:** **Basic event characteristics of BCS and FS**.

Patient no.	Vigilance	Body position	Circumstances	Impaired consciousness	Seizure duration (s)	EEG onset (ictal)	PGES	AHD	AED
1	Asleep	Laying	Spontaneous	Yes	32	Fronto-temporal (R)	No	No	LEV, LTG, CLB
2	Awake	Sitting	Triggered by music	Yes	50	Temporal (L)	No	No	LTG
3	Awake	Laying	Spontaneous	Yes	102	Generalized	No	No	LTG
4	Awake	Laying	Spontaneous	No	12	No focal onset	No	No	LTG, OXC, TPM, RFM, DZP
5	Awake	Laying	Spontaneous	Yes	94	Temporal (B)	Yes	No	OXC, LTG
6	Asleep	Laying	Spontaneous	Yes	123	Temporal-occipital (R)	No	No	No
7	Awake	Laying	Spontaneous	No	11	Temporal-occipital (L)	No	No	LTG, CLB, PER, LCM, PGB
8	Awake	Laying	Spontaneous	Yes	84	Temporal-occipital (R)	Yes	MP	LEV, LCM
9	Awake	Laying	Spontaneous	Yes	16	Temporal (R)	No	No	LEV, LCM, LTG
10	Awake	Sitting	Spontaneous	Yes	138	Generalized	Yes	No	OXC
11	Awake	Laying	Triggered by stimulation^a^	No	203	Temporal (L)	No	No	LEV, LCM
12	Awake	Laying	Spontaneous	Yes	98	Temporal-parietal (L)	No	No	LTG
13	Awake	Sitting	Spontaneous	Yes	146	Temporal-occipital (R)	No	No	No
14	Awake	Laying	Spontaneous	Yes	71	Temporal-occipital (R)	No	No	TPM
15	Awake	Laying	Spontaneous	No	13	No ictal rhythm	No	No	CBZ, OXC, VPA, CLB
16	Asleep	Laying	Spontaneous	Yes	102	Temporal (L)	No	No	No
17	Awake	Laying	Spontaneous	480	41	No focal onset	No	No	LEV, OXC
18	Awake	Laying	Spontaneous	Yes	77	Temporal (L)	No	No	No
19	Awake	Sitting	Spontaneous	No	36	No focal onset	No	No	No
20	Awake	Laying	Triggered by stimulation^a^	Yes	678	Frontal (R)	No	No	LTG, TPM
21	Asleep	Laying	Spontaneous	Yes	96	Temporal (R)	No	CAN, HCT	CBZ
22	Asleep	Laying	Spontaneous	No	32	No focal onset	No	No	LEV, ZNS, OXC
23	Awake	Laying	Spontaneous	Yes	62	No focal onset	No	No	LEV, LCM, PER
24	Awake	Laying	Spontaneous	No	40	Temporal (R)	No	No	LTG, PER
25	Awake	Laying	Spontaneous	Yes	19	No focal onset	No	No	PHB, LEV, LCM, PER
26	Awake	Laying	Spontaneous	No	27	No focal onset	No	No	No

*AED, anti-epileptic drug on the day of the seizure; AHD, antihypertensive drug on the day of the seizure; BCS, bilateral convulsive seizures; CAN, candesartan; CBZ, carbamazepine; CLB, clobazam; DZP, diazepam; ESL, eslicarbazepine; FSs, focal seizures; HCT, hydrochlorothiazide; L, left; MP, metoprolol; LCM, lacosamide, LTG, lamotrigine; LEV, levetiracetam; OXC, oxcarbazepine; PER, perampanel; PHB, phenobarbital; PGB, pregabalin; PGES, postictal generalized EEG suppression; R right; RFM, rufinamide; TPM, topiramate; ZNS, zonisamide*.

*^a^Stimulation refers to electrical stimulation of an implanted depth electrode*.

**Table 4:** **Summary of the final model (sequence method)**.

Variable	Coefficient β	95% confidence interval	*T*-value	*p*-Value
Intercept^a^	10.1^b^ ms/mm Hg	7.8−13^b^ ms/mm Hg	18.2	<0.001
Interictal time interval	99.6%	79.4−124.91%	−0.036	0.97
Postictal time interval	89.8%	67.4−119.6%	−0.755	0.45
Seizure type (BCS)	158%	95.6−261.1%	1.88	0.07
Interaction (interictal and BCS)	98.6%	63.7−152.7%	−0.06	0.95
Interaction (postictal and BCS)	33%	19−57.4%	−4.035	<0.001

*^a^Intercept refers to the reference class of the model focal seizure (FS) and preictal time interval*.

*^b^Geometric mean of baroreflex sensitivity (BRS)*.

*BCS, bilateral convulsive seizures*.

**Table 5:** **Summary of the final model (spectral method)**.

Variable	Coefficient β	95% confidence interval	*T*-value	*p*-Value
Intercept^a^	8.1^b^ ms/mm Hg	6.8−9.6^b^ ms/mm Hg	24.3	<0.001
Interictal time interval	95.5%	78.6−116.2%	−0.47	0.64
Postictal time interval	96.9%	80.4−116.8%	−0.35	0.73
Seizure type (BCS)	122.3%	86.9−172%	1.21	0.24
Interaction (interictal and BCS)	108.9%	74.7−158.8%	0.45	0.65
Interaction (postictal and BCS)	55.7%	38.9−79.7%	−3.28	0.002

*^a^Intercept refers to the reference class of the model focal seizure (FS) and preictal time interval*.

*^b^Geometric mean of baroreflex sensitivity (BRS)*.

*BCS, bilateral convulsive seizures*.

### BSR According to the Sequence Method

**Figure 1:**
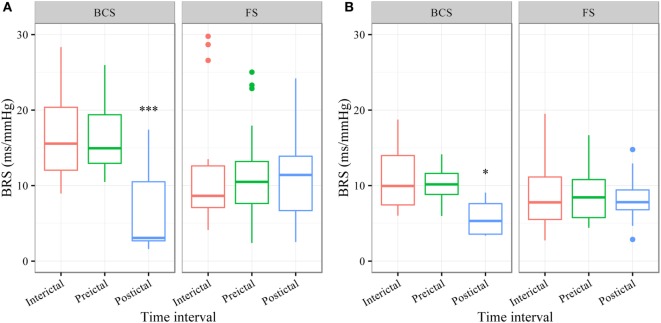
**The baroreflex sensitivity (BRS) is markedly decreased after bilateral convulsive seizures (BCS), but not after focal seizures (FSs)**. Box plots of BRS during the different time intervals for BCS and FS calculated with the sequence **(A)** and spectral method **(B)**. ***BRS calculated with the sequence method is markedly reduced during the postictal time interval compared to preictal (adjusted *p*-value < 0.0001) and interictal time interval (adjusted *p*-value < 0.0001). *BRS calculated with the spectral method is markedly reduced during the postictal time interval compared to preictal (adjusted *p*-value = 0.014) and interictal time interval (adjusted *p*-value = 0.014).

**Figure 2:**
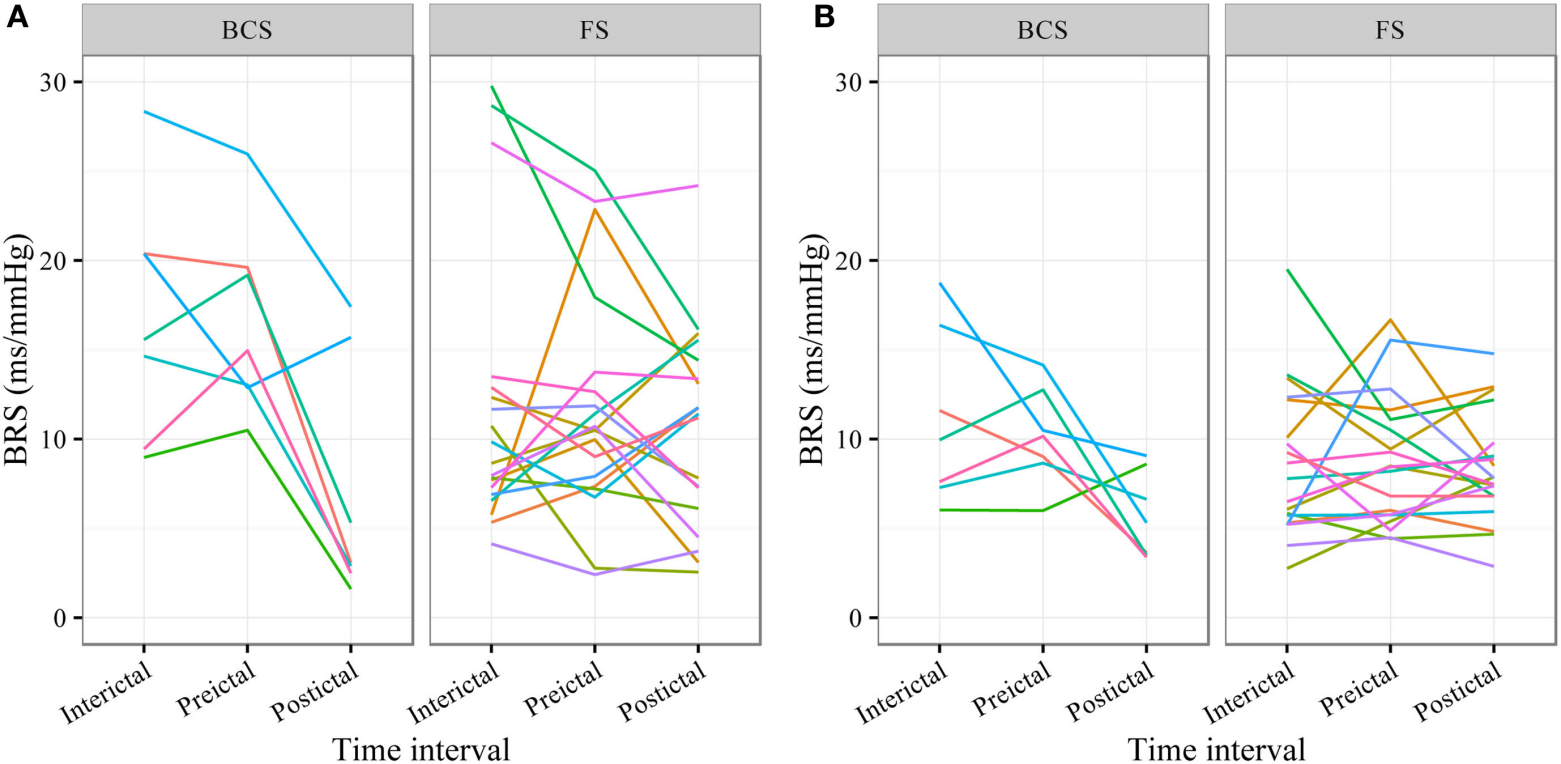
**Individual plots of baroreflex sensitivity (BRS) for each patient during the different time intervals for bilateral convulsive seizures (BCS) and focal seizures (FSs) calculated with the sequence (A) and spectral method (B)**.

In BCS (*n* = 7), BRS significantly dropped from a preictal value of 15.0 ms/mm Hg (IQR 13.0−19.4, adjusted *p*-value < 0.0001) and interictal value of 15.6 ms/mm Hg (IQR 12.0−20.4, adjusted *p*-value < 0.0001) to a postictal value of 3.1 ms/mm Hg (IQR 2.7−10.5). Although not statistically significant, BCS with PGES (*n* = 3) tended to be associated with a lower postictal BRS as compared to BCS without PGES (*n* = 4, adjusted *p*-value = 0.10). In FS (*n* = 19), no significant difference was found for the preictal BRS of 10.5 ms/mm Hg (IQR 7.6−13.2) as compared to the interictal BRS of 8.6 ms/mm Hg (IQR 7.1−12.6) or the postictal BRS of 11.4 ms/mm Hg (IQR 6.7−13.9). Figure 1A (left panel) summarizes the BRS values calculated with the sequence method in patients with BCS and FS. Figure 2A (left panel) shows the individual profiles of the patients.

### BSR According to the Spectral Method

In BCS (*n* = 7), BRS significantly decreased from 10.2 ms/mm Hg (IQR 8.8−11.6, adjusted *p*-value = 0.014) preictally and 10.0 ms/mm Hg (IQR 7.4−14.0, adjusted *p*-value = 0.014) interictally to 5.3 ms/mm Hg (IQR 3.6−7.6) postictally. BRS in BCS with or without PGES did not differ significantly during the postictal time interval (adjusted *p*-value = 0.24). In FS (*n* = 19), postictal BRS (7.8 ms/mm Hg, IQR 6.8−9.4) was not different from preictal (8.4 ms/mm Hg, IRQ 5.8−10.8) or interictal (7.8 ms/mm Hg, IQR 5.5−11.1) values. Figure 1B (right panel) summarizes the BRS values calculated with the spectral method in patients with BCS and FS. Figure 2B (right panel) shows the individual profiles of the patients.

## Discussion

In summary, we found that in contrast to FS, BRS is markedly impaired in the early postictal period following BCS, suggesting that BCS lead to substantial postictal autonomic dysfunction.

### Potential Mechanisms of Seizure-Related Modulation of BP and BRS

The mechanisms of how epileptic seizures affect systemic BP and BRS are not fully understood (1). The anatomic basis consists of the sympathetic and parasympathetic branches of the autonomic nervous system, which regulates BP *via* their effects on HR, cardiac output, and total peripheral resistance. The central pathway of the baroreflex involves several brain structures (6). The nucleus tractus solitarius receives the afferent input of baroreceptors. From there the parasympathetic branch is processed through the nucleus ambiguous and finally inhibits the sinus node to reduce HR and cardiac output. The sympathetic pathway is processed through the caudal ventrolateral medulla, rostral ventrolateral medulla and preganglionic sympathetic neurons (5). From there the sympathetic outflow mainly increases total peripheral resistance through vasoconstriction of muscle, renal, and mesenteric blood vessels. In addition, subordinate centers continuously modulate the baroreflex arc.

In FS, BP, and HR both display a transient increase with a similar time course, probably due to an increase of the sympathetic activity through stimulation of central autonomic centers by epileptic activity (4). In BCS, systemic BP appears to display at least two different patterns. The first pattern is characterized by a simultaneous increase of systemic BP and HR during the seizure (4, 18, 19). The second pattern consists of a biphasic behavior of BP with an initial increase followed by a drop (4, 19). The HR is commonly accelerated during BCS (4, 20). Importantly, the time course of seizure-related HR and BP alterations are different. While the HR remains elevated up to 30 min after seizure termination, systemic BP is only slightly elevated by about 15% in the early postictal phase and returns to baseline levels or even drops slightly below preictal values within 5 min after seizures cessation (4, 20). This opposed behavior of HR and systemic BP in spite of a considerable seizure-related release of catecholamines is somewhat surprising, but most likely caused by an immediate muscular hyperemia that commonly follows exercise of skeletal muscles which, in turn, leads to a decreased systemic vascular resistance and ultimately to a drop in systemic BP (21). To counteract the drop in systemic vascular resistance and systemic BP, HR may be increased *via* the arterial baroreflex, which could partially explain the increase of HR following BCS (4, 20). The abovementioned seizure-related local and systemic metabolic effects, however, may also impair BRS to some extent.

An alternative explanation for impaired BRS after BCS is an inhibitory effect on central autonomic centers due to exhausted or suppressed brain activity (18). For example, BCS are frequently associated with a PGES (22, 23). In one case report, postictal hypotension and PGES were associated (18). However, in a larger study, no association between postictal BP changes and PGES was found (4). In the present study, BCS (assessed with the sequence method) with PGES tended to have lower BRS during the postictal period than BCS without PGES. This result has to be considered with caution because of the small sample size and because this finding could not be reproduced by the BRS analysis according to the spectral method.

### Potential Clinical Implications of Impaired BRS following BCS

The supply of metabolites and oxygen depends on the blood perfusion, which is directly linked to the systemic BP and tightly regulated in most organs (5). The baroreflex continuously stabilizes systemic BP and prevents excessive BP rises or falls (24). A *permanently decreased* BRS has been observed in many chronic diseases including diabetes mellitus, obesity, hypertension, and coronary artery disease and predicts a poor prognostic outcome (5). For instance, patients with myocardial infarction or congestive heart failure are at higher risk of sudden cardiac death (13). *Acute non-selective* baroreflex failure usually leads to hypertensive crisis or fluctuating hypertension (25). In particular subjects with selective baroreflex failure in whom the efferent parasympathetic pathway to the heart remains intact, however, can also suffer from hypotensive episodes (26).

Our study indicates a marked *acute* autonomic dysfunction after BCS. Most SUDEP cases are probably caused by a cardiorespiratory failure in the early postictal phase following BCS (3). Importantly, a recent case report described a significant drop of systemic BP in the aftermath of a BCS (18). If severe postictal hypotension in conjunction with an impaired BRS were one of the mechanisms facilitating SUDEP, these events may be prevented by an early cardiopulmonary resuscitation. Indeed, in seven out of nine near-SUDEP cases, cardiopulmonary resuscitation was successfully carried out within the first 3 min of seizure cessation (3). In contrast, in 8 of 12 SUDEP cases, cardiopulmonary resuscitation was initiated later than 10 min after seizure termination. Therefore, it seems advisable that systemic BP, HR, and breathing rate should be monitored in the early postictal period if possible, e.g., in video-EEG telemetry units during assessment for epilepsy surgery.

### Study Limitations

Our study comes with some limitations. As we retrospectively selected patients, this study has an observational character and may be subject to various confounders. Firstly, because of the relatively small sample size, we may have overlooked smaller effects of an influencing factor in our patient sample. For example, because only three BCS were followed by PGES, the model may have failed to detect a significant effect on BRS. Second, patients with lesions in areas which affect autonomic regulatory sites may have additional or stronger postictal alterations of BRS. Due to the small sample size of patients with these lesions and epilepsy-type matched patients without such lesions, this question could not be directly addressed in our study. Third, we cannot rule out possible effects of anticonvulsant drugs or the withdrawal of anticonvulsant drugs on BRS. These putative effects, however, should be the same for both FS and BCS, strengthening the finding of differential effects on BSR depending on the seizure type. Fourth, confounders such as variable body position, hypertension, or vigilance state may have an impact on the BRS. However, we assessed this issue by including these variables into the model without apparent significant effect on BRS. In addition, we verified our findings comparing the BRS of postictal interval not only with the BRS of the preictal but also with the BRS of the interictal time interval. Fifth, spontaneous BRS methods have some limitations compared to the gold standard, the phenylephrine method (27). For example, the measures do not always correlate well with pharmacological methods (28). However, the phenylephrine method has also several drawbacks. For instance, it demonstrates a large intra-individual variability of response if it is repeated in the same individual (29). In addition, phenylephrine induces changes in venous compliance and venous return and may activate the afferent branch of baroreflex pathways regardless of the increase in systemic BP (29). Furthermore, the pharmacological methods involve patients to risks, because they are invasive procedures with all the potential harmful effects. We therefore chose a spontaneous baroreflex method, whose baroreflex nature has been demonstrated on an animal model (30). This method was also validated against the phenylephrine method in healthy subjects (31). In addition, we reproduced our results using a different technique, the spectral method, thereby strengthening our findings and conclusions (12). Finally, our interictal and preictal BRS values were in a similar range than that of healthy subjects reported in previous studies, which underlines the reliability of our estimates (12, 14, 32).

## Conclusion

We found that postictal BRS is markedly reduced after BCS in patients with focal epilepsy, providing additional evidence for severe autonomic dysfunction related to BCS. Our findings might be linked to cardiovascular failure facilitating SUDEP. Further studies with larger sample sizes are needed to verify our results and to deepen our understanding of periictal modulation of systematic BP and BRS.

## Ethics Statement

The study was approved by the local medical ethical committee of the University of Bonn. Each patient had signed an informed consent in accordance with the Declaration of Helsinki.

## Author Contributions

KH has conducted the recordings, contributed to the study design, the data analysis, and writing of the manuscript. CE has contributed to interpretation of the data and has revised the manuscript critically for important intellectual content. RS has contributed to the study design, the data analysis, and writing of the manuscript.

## Conflict of Interest Statement

The authors declare that the research was conducted in the absence of any commercial or financial relationships that could be construed as a potential conflict of interest. KH has received support from Cyberonics, UCB Pharma, Grupo Juste, and EISAI. CE has served as a paid consultant for UCB Pharma, Desitin, and Pfizer. He is an employee of the Life and Brain Institute Bonn. RS has received fees as speaker or consultant from Bial, Cyberonics, Desitin, EISAI, Novartis, and UCB Pharma.
